# Understanding the Reactivity of *N*-Heptane Blended with Ethanol or Ethyl Acetate

**DOI:** 10.1021/acsomega.4c10828

**Published:** 2025-05-01

**Authors:** Mathias Grunewald, Marcel Neumann, Marius Hofmeister, Adrian Nolte, Stefan Pischinger, Katharina Schmitz, Karl Alexander Heufer, Reinhold Kneer

**Affiliations:** † RWTH Aachen University - Institute of Heat and Mass Transfer (WSA), Aachen 52062, Germany; ‡ RWTH Aachen University - Chair of Thermodynamics of Mobile Energy Conversion Systems (TME), Aachen 52072, Germany; § RWTH Aachen University - Institute for Fluid Power Drives and Systems (ifas), Aachen 52056, Germany; ∥ RWTH Aachen University - Chair of High Pressure Gas Dynamics (HGD), Aachen 52074, Germany

## Abstract

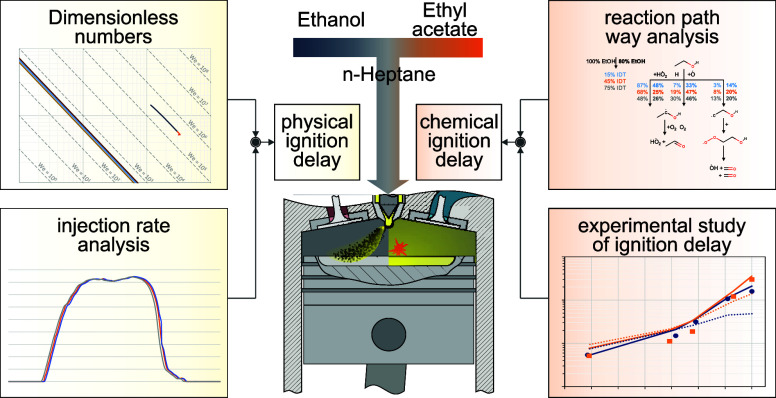

Fuel properties (viscosity, density, surface tension, ignition delay times) of binary mixtures containing a highly reactive fuel (*n*-heptane) and a low-reactive fuel (ethanol or ethyl acetate) are investigated in this study. For certain mixing ratios, the ethanol blend is found to exhibit longer ignition delay times after injection than the ethyl acetate blend, particularly noting that pure ethanol shows shorter ignition delay times than pure ethyl acetate. To explore the underlying causes, a comprehensive analysis is conducted, focusing on injection dynamics, mixture formation, and the chemical mechanisms leading up to ignition. Experiments on physical fluid properties, including viscosity, density, and surface tension, are performed to assess potential fluid mechanical effects on ignition delay times, with these properties fitted to existing mixing rules. Theoretical ignition delay times for different mixing ratios are calculated using a kinetic model, while experiments using a rapid compression machine provide insights into the purely chemical ignition delay for specific mixture ratios across various temperatures. Additionally, a rate-of-production analysis is conducted to offer a deeper understanding of the changes in reactivity observed in these fuel blends. Through this analysis, it becomes apparent that the change in reactivity is due to a change in the reaction pathways for ethyl acetate.

## Introduction

In response to the challenges posed by climate change, the shift away from fossil fuels, and the increasing demands on modern combustion engines, there is a growing focus on the intensive investigation of a wide range of alternative fuels.^1−3^ This endeavor involves evaluating a broad spectrum of candidates, many of which fall short of existing standards for traditional gasoline or diesel fuels. Consequently, there is a drive to modify these alternatives to achieve optimal combustion properties while ensuring compatibility with current engine materials. Another requirement for future fuels is a synthesis path that is as sustainable as possible.^4^ One possibility to tailor properties is blending suitable biohybrid fuels to achieve specific desirable characteristics needed for combustion, which may be expressed through characteristic numbers such as Research Octane Number (RON) or Cetane number (CN).^4,5^ Investigations are being carried out to determine the effects that blending has on different physical fuel properties and the reactivity.^6−8^ Within the framework of the excellence cluster Fuel Science Center (FSC) at RWTH Aachen University, several promising fuel candidates have already been selected in the past.^4,9,10^ Fuel blends composed of various proportions of *n*-heptane (representing high-reactivity fuel) and ethanol or ethyl acetate (respectively both representing low-reactivity fuels) have emerged as promising candidates for enhancing combustion efficiency and reducing emissions.^11^ In most cases, the optimization of blend composition is performed using simplified models, such as those predicting reactivity based on RON or CN values. Properties of binary fuel blends, such as viscosity, density, and surface tension, can be described accurately by existing mixing rules.^12−14^ In general, depending on the mixing ratio, steady, strictly monotonic curves of the investigated property can be expected. These parameters are hereby determined by linear interpolation of the neat fuel data.^9^ Assuming there are three components A, B, and C, where *RON*_*A*_ > *RON*_*B*_ > *RON*_*C*_, these blend models would predict that a proportionally equal mixture will always result in *RON*_*A*/*C*_ > *RON*_*B*/*C*_. Fuel blends, consisting of highly reactive (HRF) and low-reactive fuels (LRF) were investigated with regard to their ignition delay times in an Advanced Fuel Ignition Analyzer (AFIDA).^15^

**Figure 1:**
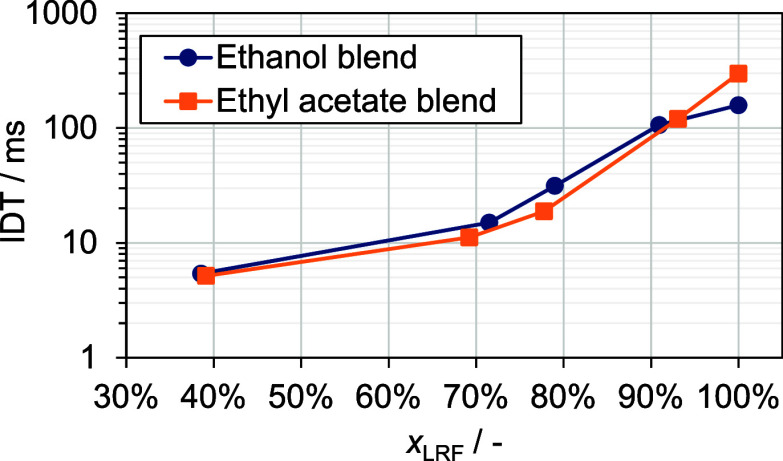
Ignition delay times (IDT) for varying fuel blends (the percentage values on the *x*-axis represent the proportion of the low-reactive fuel).

These experiments enabled insights into the reactivity of different HRF/LRF blends and it was observed that for certain blending ratios, the aforementioned hypothesis regarding the reactivity of fuel mixtures does not hold (see Figure 1). For mixing ratios of 70 vol % and 80 vol %, the ethanol blend exhibits longer ignition delay times compared to the ethyl acetate blend, while pure ethanol has shorter ignition delay times than pure ethyl acetate.^15^ In the subsequent sections, a comprehensive examination of the foundational causes will be presented, focusing on the investigation of injection dynamics, mixture formation, and the chemical mechanisms preceding ignition. To investigate possible fluid mechanical effects on the ignition delay times, experiments on the physical fluid properties as well as the resulting injection behavior are conducted. The results for viscosity, density, and surface tension are fitted to existing mixing rules. To gain insights into the underlying chemistry causing the change in reactivity, a kinetic model is used to calculate theoretical ignition delay times for different mixing ratios. Furthermore, experiments using a rapid compression machine (RCM) enable the investigation of the purely chemical ignition delay time for a single mixture ratio (80 mol % LRF) at different temperatures. Additionally, a rate-of-production analysis is conducted to gain deeper insights into the reactivity changes observed in fuel blends.

## Methodology

The present experimental setup seeks to comprehensively examine the combustion and physical properties of fuel blends, composed of various proportions of *n*-heptane (representing high-reactivity fuel) and ethanol or ethyl acetate (respectively both representing low reactivity fuels), with particular emphasis on their ignition characteristics, injection behavior, as well as viscosity, density, and surface tension. All experiments within this study revolve around the systematic preparation of fuel blends by incrementally mixing *n*-heptane (RON = 0), and ethanol or ethyl acetate in steps of 10 volume-percent going from pure high-reactivity (*n*-heptane) to pure low-reactivity fuel (ethanol, ethyl acetate with RON = 109 and RON = 118). This deliberate variation in the composition allows for a detailed exploration of the effects of different fuel combinations on combustion and the previously mentioned selected physical properties. To achieve this, a range of experimental techniques and apparatuses have been employed. In this section, these experimental setups including measurement equipment and operating conditions are described.

### Investigation of Ignition Delay Time

The ignition delay time (IDT) is characterized as the time interval from the attainment of conditions enabling ignition to the point of autoignition itself. The ignition experiments were conducted with an Advanced Fuel Ignition Delay Analyzer (AFIDA, including transport processed) and a Rapid Compression Machine (decoupled from transport processes). In addition, both experiments were modeled through zero-dimensional (0-D) approaches.

#### Advanced Fuel Ignition Fuel Ignition Delay Analyzer

An advanced fuel ignition delay analyzer (AFIDA) provided by ASG Analytik-Service GmbH was used to measure the ignition delay time (IDT).^16^ The AFIDA is a fully automated ignition delay analyzer. In the AFIDA, injection and mixture formation are included in the ignition delay time as well as the purely chemical ignition delay. The analyzer utilizes a Constant Volume Combustion Chamber (CVCC) and operates under the standard conditions (853 K, 17.5 bar) for determining the derived cetane number.^17^ The fuel injection pressure was set to 1000 bar and the injector trigger duration was set to 3 ms to ensure a self-ignitable air-fuel ratio λ for long IDTs. The amount of fuel injected was measured following the method used by Luecke et al.,^18^ which involves conducting two consecutive measurements with the same fuel. The injected fuel amount can be determined by weighing the vial before and after the experiment as well as the fuel waste bin.

#### Rapid Compression Machine

**Figure 2:**
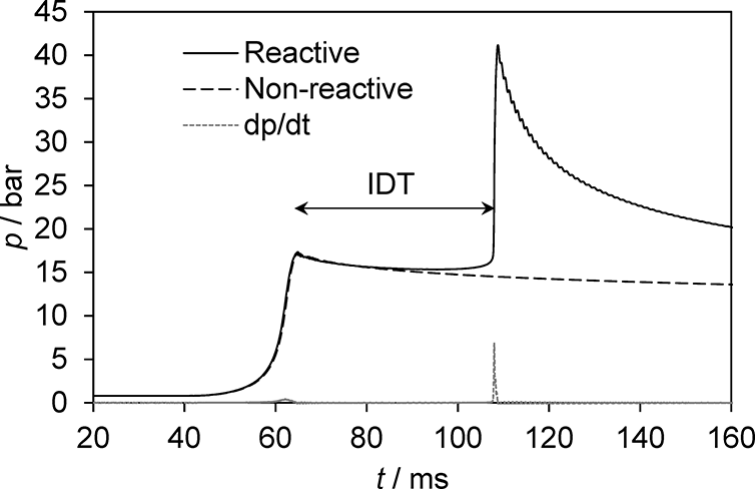
Exemplary experiment with 80 vol % ethanol, 20 vol % *n*-heptane, ϕ = 0.5, 17.2 bar, and 839 K.

To investigate the ignition delay time decoupled from any transport processes (chemical ignition delay time), experiments were carried out in a Rapid Compression Machine (RCM) as described by Burke et al.^19^ In a RCM, a mixture of fuel, oxidizer and bath gas is compressed to high pressure and temperature by a piston, leading to autoignition after a characteristic ignition delay time. The RCM used in these experiments consists of a single-piston configuration with a flexible compression ratio of 9 to 32. The piston system is accelerated by pneumatic pressure, while the stopping process is controlled by a hydraulic section. The premixing was conducted in a heated manifold by controlling the partial pressures of the corresponding components. For the static pressure measurements, two sensors by the manufacturer STS have been used (PTM/RS485 0.5 and 5.0 bar). The dynamic pressure during the experiments was recorded with a recessed Kistler sensor (6125CU20). Initial temperatures were homogeneously set to 75 ^◦^C with a well-proven heating system.^19^ For each experimental condition, nonreactive experiments have been performed by replacing O_2_ by N_2_. From the experimental nonreactive pressure trace, volume ratio profiles have been derived in order to model the RCM with associated facility effects. All volume ratio profiles are given in the Supporting Information. An exemplary pressure trace is given in Figure 2 with the definition of the ignition delay time between the end of compression time and the time of highest-pressure slope. Uncertainties in the ignition delay time for the used rapid compression machine have been estimated by Burke et al.^19^ to be ± 20%. However, a more recent and detailed uncertainty evaluation by Preußker et al.^20^ resulted in an uncertainty of ± 1% for the end-of-compression temperature and an experimental scatter of the ignition delay time in the order of ±15%. These values are displayed in the following figures as error bars for the rapid compression machine experiments. However, the temperature uncertainty combined with the experimental scatter and other nonmeasurable factors sums up to an estimated total uncertainty in the ignition delay time of around ±25–30%.^20^

### Investigation of Physical Fuel Properties

Furthermore, the investigation encompasses the physical fluid properties of the fuel mixtures, including viscosity, density, and surface tension, alongside the ignition delay time. To achieve this, mixtures were systematically prepared with the *n*-heptane content increasing in increments of 20 volume percent. The fuels were also examined in their pure liquid forms. The preparation of the mixtures took place in a climatic chamber maintained at 20 °C, utilizing a glass pipet with a resolution of 0.1 mL. All subsequent measurements were conducted at a constant temperature of 20 ^◦^C.

#### Determination of Viscosity

The kinematic viscosity ν of the fuel mixtures is determined using an Ubbelohde viscometer (Fisher SI Analytics) in accordance with DIN 51562.^21^ Temperature regulation is achieved through a thermostat with a precision of 0.01 K. During the assessment, the viscometer is immersed in the thermostat, which is filled with a heating medium, to ensure that the sample liquid and the capillary attain the temperature of the surrounding medium. The viscometer is left within the thermostat for 30 min post sample liquid introduction to achieve temperature equilibrium. Initially, two preliminary trials are conducted to cleanse the capillary, followed by five principal measurements. The outcomes of the preliminary trials are excluded from the analysis, whereas the average of the principal measurements is computed for subsequent evaluation. Due to the discrepancy between the viscometers’ measurement range, as specified by DIN 51562,^21^ and the viscosity of the mixtures, an appropriate viscometer is selected for each sample. In this investigation, a class 0 viscometer is employed for each sample under study.

#### Determination of Density

**Figure 3:**
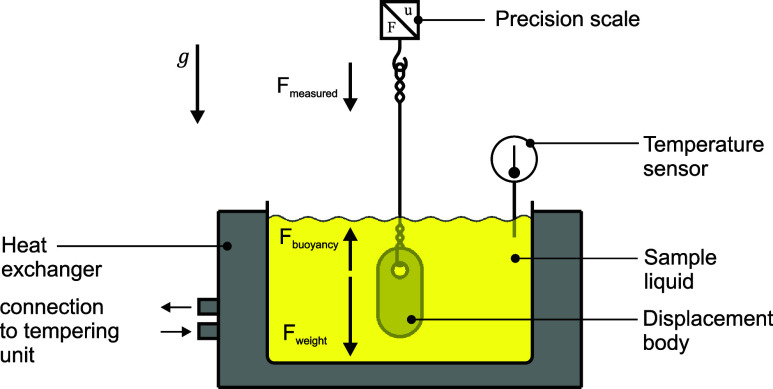
Test setup for the determination of liquid density.

For the determination of the fuel density ρ_*liq*_, the measurement setup depicted in Figure 3 is used.

The setup mainly consists of a displacement body, a wire connected to a tension scale, and a vessel filled with the sample liquid. At the beginning of the experiment, the wire is immersed into the sample liquid and the tension scale is set to zero. Subsequently, the displacement body is attached to the wire. The resulting weight is recorded via the tension scale. To calculate the fuel density, an equilibrium of forces consisting of the measured weight force, the weight force of the displacement body, and its buoyancy force is established. The corresponding equation depending on mass *m*_*DB*_ and volume *V*_*DB*_ of the displacement body as well as the gravitational constant g and the liquid density ρ_*liq*_ is shown in eq 1.
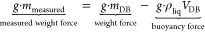
1

Through elimination of the gravitational constant, eq 1 can be rearranged and the liquid density now given by the difference of the mass of the displacement body and the measured mass divided by the volume of the displacement body (eq 2).
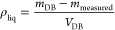
2

Since the volume and mass of the displacement body are known, the liquid density ρ_liq_ only depends on the mass measured. To keep the temperature of the sample liquid at a constant level, the vessel is placed in a heat exchanger, which is connected to a tempering unit. The used tempering unit has a temperature constancy of 0.02 K.

#### Determination of Surface Tension

The surface tension σ of the mixtures is measured by the Du Noüy ring method according to DIN EN 14210.^22^ The ring diameter is 19.1 mm and the diameter of the wire is 0.4 mm. The ring is manufactured of an alloy, which consists of 50% iridium and 50% platinum. For maintaining a constant temperature during the measurement, the heat exchanger and tempering unit previously used for density determination were implemented.

#### Investigation of Injection Characteristics

To characterize the injection process, evaluations were performed using an injection progress indicator as described in a 1964 report by Robert Bosch GmbH.^23^ This system facilitates the determination of both the total volume of fuel injected and the rate of injection (quantity of fuel injected per unit time). The injection progress indicator employs an indirect measurement principle, meaning that it gauges specific physical parameters from which the desired variable is subsequently computed, rather than directly measuring the sought-after quantity.^24^ In this specific scenario, the measured parameter is the time-varying pressure curve within the measuring tube, from which the mass flow curve is subsequently calculated. *n*-Heptane, ethyl acetate (EA) and ethanol, as well as their respective blends, were tested in this apparatus. The conditions for injection were set at 17.5 bar counter pressure and an injection pressure of 1000 bar, as well as an injection time of 1.5 ms, in order to enable comparison with the AFIDA. Since the injector used in the AFIDA cannot be used for investigations on the injection progress indicator, a generic automotive injector was used to investigate the injection processes.

#### Evaluation of Dimensionless Numbers

For the characterization of the spray behavior, the Ohnesorge diagram is used. To create the Ohnesorge diagram, the Reynolds numbers, which relate inertial forces and friction forces on the liquid, are plotted on the *x*-axis. The Ohnesorge number puts friction forces and the root of inertia and surface forces in a relation to each other and is plotted on the *y*-axis. For constant Weber numbers, diagonal curves are obtained, and a comparable degree of atomization can be expected. The underlying formulas for calculating the individual dimensionless numbers are listed in Table 1. For increasing Reynolds and Ohnesorge numbers, droplet size decreases. In case of automotive applications, a small droplet size and therefore a high degree of atomization is desirable. In literature, different gas Weber numbers are reported for which atomization is to be expected. According to Miesse,^25^ for example, atomization begins at a gas Weber number larger than 40.3, and larger than 13 according to Ranz.^26^ In this study, a Weber number larger than 40.3 is used. The dimensionless numbers are calculated based on the fitted physical fluid properties discussed before, nozzle diameter and nozzle outlet velocity. The physical property data used for the calculation of dimensionless numbers are derived from specific mixing models: the Redlich–Kister model^12^ for kinematic viscosity, a linear approach for density, and the Connors-Wright model^13^ for surface tension. Since the exact nozzle outlet velocity is unknown, it is estimated by the Bernoulli principle shown in eq 3. The indices indicate the different densities, pressures, and velocities at the nozzle inlet and outlet, where 1 corresponds to the inlet and 2 to the outlet.


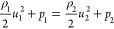
3

It is assumed that the velocity at the nozzle inlet is negligible compared to the velocity at the outlet and that the outlet pressure only has a minor influence on the outlet density. Therefore, the outlet velocity only depends on the pressure difference and outlet density, and can be calculated according to eq 4.
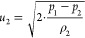
4

### Kinetic Model

Enhanced understanding of the kinetic disparities between the oxidation of pure components and that of their corresponding blends can be achieved through simulation techniques. For this purpose, a new kinetic model has been developed based on the combination of two preexisting models.^27^ The choice of NUIGMech1.1 as the foundational mechanism is justified by its inclusion of a well-validated ethanol model and a comprehensive *n*-heptane mechanism, making it apt for such analytical pursuits. Additionally, the revised ethyl acetate model by Morsch et al.,^3^ which employs a reduced version of the NUIGMech as its underlying framework, was integrated into the existing NUIG mechanism. The resulting model contains 2801 species and 11609 reactions and is integrated in the Supporting Information. The simulations presented in this study were executed using the Cantera software package, employing a zero-dimensional (0-D) approach.^28^

## Results and Discussion

The structure of the following section is organized as follows: First, the findings related to ignition delay times are presented. This is followed by an assessment of the physical properties of the fuels and injection characteristics. The section concludes with the classification and explanation of observed anomalies, using simulation analysis to elucidate reaction pathways.

### Influence of Mixing Ratios on Ignition Delay Time

In this section, the results for different methods of determining the ignition delay times will be discussed.

#### Theoretical Ignition Delay of Pure Components

**Figure 4:**
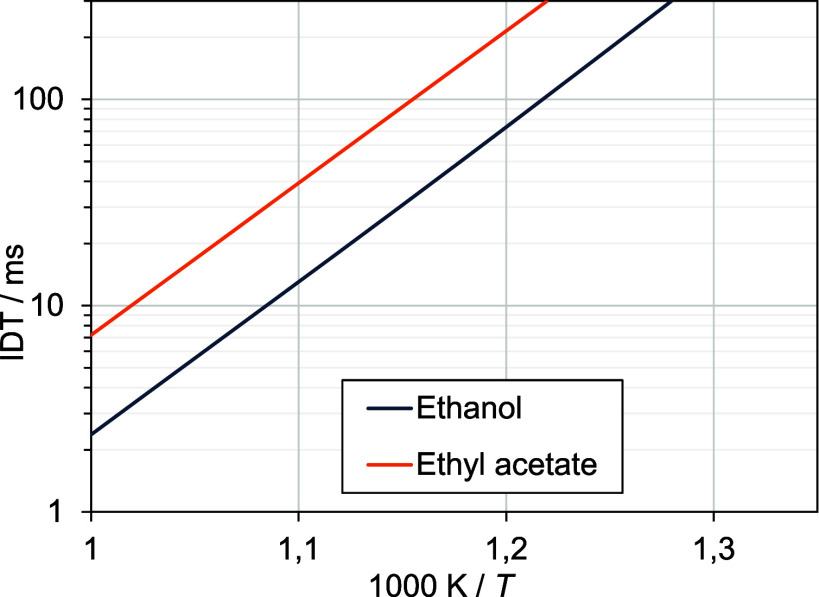
Results of a constant volume simulation at 17.5 bar and ϕ = 0.5 in air.

To facilitate a comparative analysis of different blends, it is advisable to initially examine the reactivity of the pure components. For this purpose, constant volume simulations were carried out at 17.5 bar and an equivalence ratio (ϕ) of 0.5 in air. Figure 4

The distinct Arrhenius-type behavior of both fuels is clearly observed. Moreover, the autoignition of ethanol is consistently faster across the entire range of simulated temperatures, resulting in ignition delay times (IDTs) for ethyl acetate that are approximately 200% longer.

#### Influence of Mixing Ratios on the Physical Ignition Delay Time

**Figure 5:**
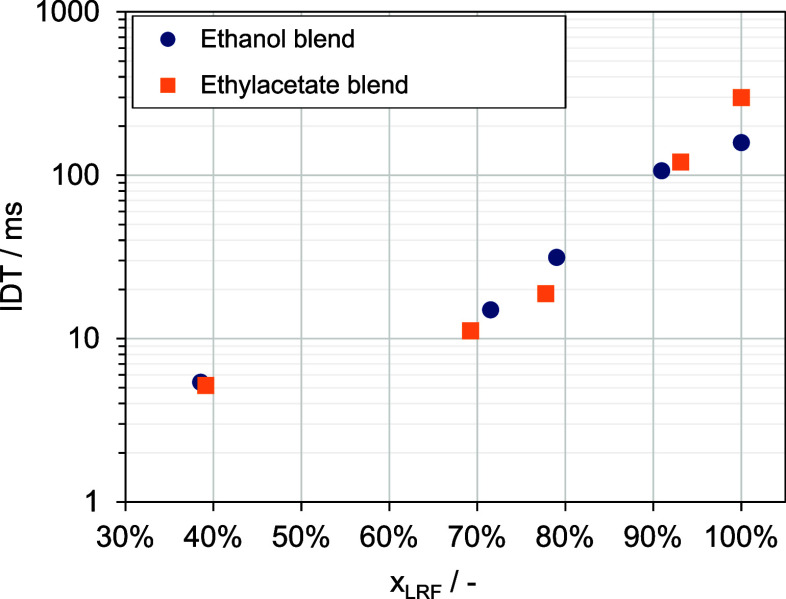
AFIDA ignition delay times at 17.5 bar, 853 K, and an injection duration of 3 ms.

Using the AFIDA, the ignition delay times were obtained for five different mixing ratios. The IDTs across these five blending ratios, with an injection duration of 3 ms at 853 K and 17.5 bar (symbols), are depicted in Figure 5. In its unblended state, ethanol exhibits a significantly higher reactivity with an IDT of 158 ms, compared to ethyl acetate’s IDT of 298 ms. Notably, introducing a mere 10 vol % (equals *x*_*LRF*_ = 90%) of *n*-heptane to each component results in equalized reactivity levels between the ethyl acetate and ethanol blends. As the *n*-heptane proportion increases to 20 vol %, a reversal in reactivity occurs, with the ethyl acetate blend becoming more reactive. At this blend ratio, an IDT of 18 ms is recorded for the ethyl acetate blend, in contrast to the ethanol blend’s IDT of 31 ms. With further increase to 30 vol % *n*-heptane, the reactivity between the two blends begins to converge once again. Ultimately, at higher *n*-heptane concentrations around 60 vol %, the reactivity difference between the blends becomes indiscernible.

**Figure 6:**
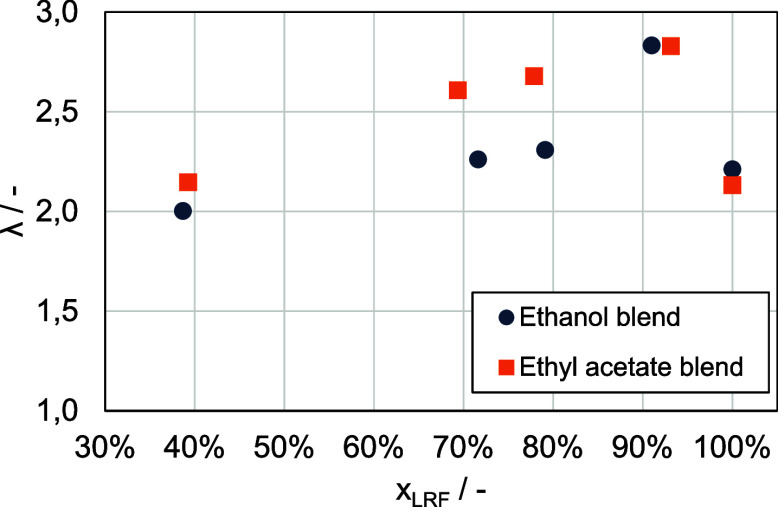
AFIDA air–fuel ratio at 17.5 bar, 853 K, and an injection duration of 3 ms.

The global λ values within the CVCC exhibit only marginal differences for low- and high-LRF shares, as well as for pure ethanol and EA, as depicted in Figure 6. The blends around 70 and 80 vol % show a stronger difference in the global λ of about 0.3. This is not considered to be significant regarding the IDT behavior, since the global λ is already larger than 2.

**Figure 7:**
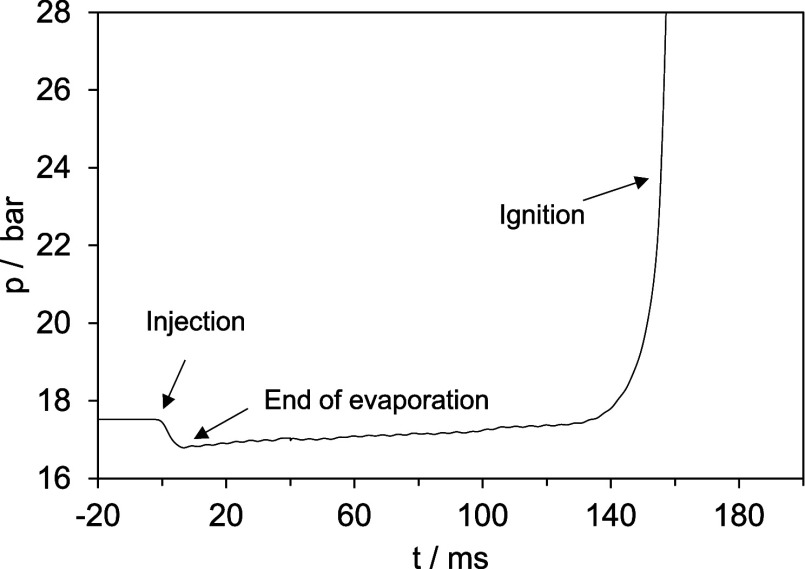
AFIDA pressure trace of neat ethanol/air mixture with 3 ms injection time.

To check if the flip in reactivity is due to the mixing behavior or due to kinetic reasons, 0-D simulations have been performed. The 0-D approach is especially applicable for long IDTs but can suffer from mixture inhomogeneities for short IDTs. For example, Luecke et al.^18^ conducted CFD simulations for the iso-octane injection in an AFIDA, with a global equivalence ratio of 1.2 and an injection time of 4 ms. They found that after 30 ms, the entire reactor is well-mixed, making transport processes negligible at this point. In this study, the AFIDA experiments used a fuel-lean global equivalence ratio of 0.35 to 0.50 with an injection time of 3 ms. Therefore, an even faster mixing time is expected. Nevertheless, the fuel injection has to be considered in the simulations as it significantly changes the measured initial temperature of the reactor. Especially the ethanol experiments should strongly be affected by the high heat of vaporization. Here, a consistent approach was applied to determine a homogeneous reactor temperature after injection. This is done through the known volume *V* of the reactor chamber and the ideal gas law:
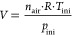
5where *n*_*air*_ is the molar amount of air in the reactor before fuel injection, *T*_*ini*_ the measured static reactor temperature, *p*_*ini*_ the initial pressure, and R the ideal gas constant. Looking at the pressure trace of Figure 7, a clear pressure drop after the injection due to the cooling effect of the lower fuel temperature and the fuel evaporation can be observed. With this measured pressure after injection *p*_*inj*_, the definition of the reactor volume V, and the ideal gas law, the temperature after fuel injection and evaporation *T*_*inj*_ can be determined by eq 6:
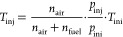
6where *n*_fuel_ is the molar amount of injection fuel and *p*_inj_ is the pressure after the end of the evaporation process. Consequently, the temperature following fuel injection is only dependent on measured values *p*_*inj*_, *p*_*ini*_, *T*_*ini*_, and the ratio of air molecules *n*_*air*_ to the total amount of molecules *n_air_* + *n*_*fuel*_ in the reactor after fuel injection. This molecular ratio is directly correlated with the global equivalence ratio, calculated from the injected fuel mass and the initial air mass in the reactor, potentially leading to considerably reduced reactor temperatures.

**Figure 8:**
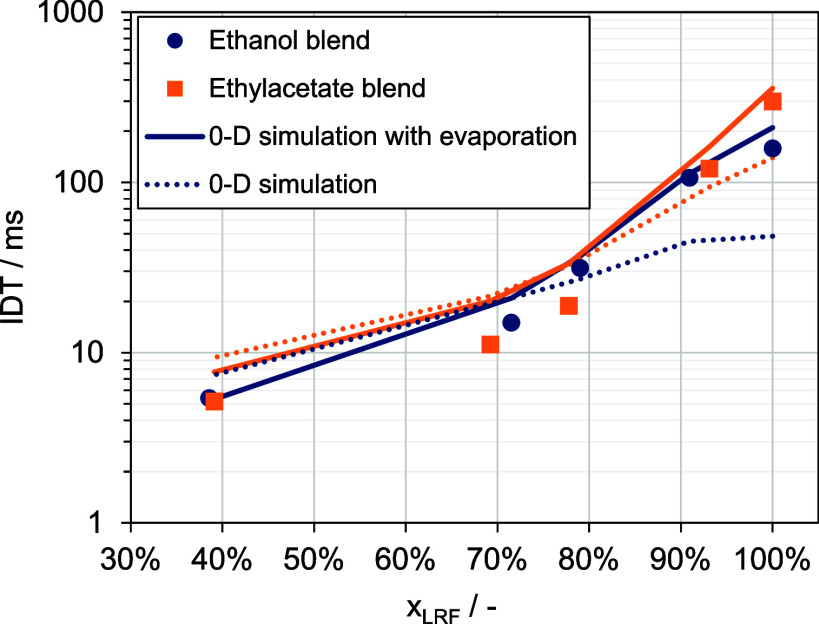
AFIDA ignition delay times at 17.5 bar, 853 K, and an injection trigger duration of 3 ms complemented by modeled results.

For instance, injecting pure ethanol into a reactor initially at 853 K results in a postinjection temperature of 797 K, considering the observed pressure drop illustrated Figure 7. This scenario was also evaluated using the van der Waals equation for real gases, yielding a temperature of 794 K. Given the minor discrepancy (3 K) falls within the methodological uncertainty, the ideal gas law was preferred for subsequent analysis. To assess the impact of these modified reactor temperatures, zero-dimensional (0-D) simulations were conducted at the initial 853 K temperature (represented by dotted lines in Figure 8) and temperatures calculated using the aforementioned approach (shown as solid lines in Figure 8).

Simulations with the adjusted initial temperatures showed excellent concordance with experimental data for 100 vol % and 90 vol % low-reactive fuel fractions. However, using a reactor temperature of 853 K significantly underpredicted the IDTs. At 80 vol % ethanol, the IDT prediction is still in accordance with the measurements but starts to overpredict the IDT at around 70 vol % ethanol. The corresponding experimental IDT for the latter case is 15 ms. At this point, it is concluded that the assumption of an ideally mixed reactor begins to fail. For ethyl acetate blends, overprediction commences at an 80 vol % fraction, though the overall trend remains accurately depicted. This discrepancy may originate from the unvalidated ethyl acetate submechanism in lean-fuel mixtures or fuel blends. To ensure model reliability, further validation through RCM experiments is essential, which will be discussed in the ensuing section.

#### Influence of the Mixing Ratio on the Chemical Ignition Delay Time

**Figure 9:**
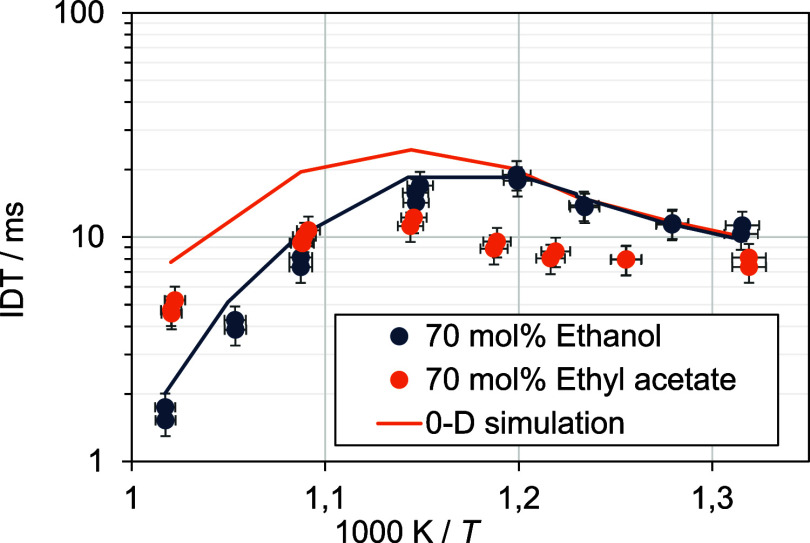
RCM ignition delay times at ϕ = 0.5, 17.5 bar, and air-like dilution for blends with 30 vol % *n*-heptane.

**Figure 10:**
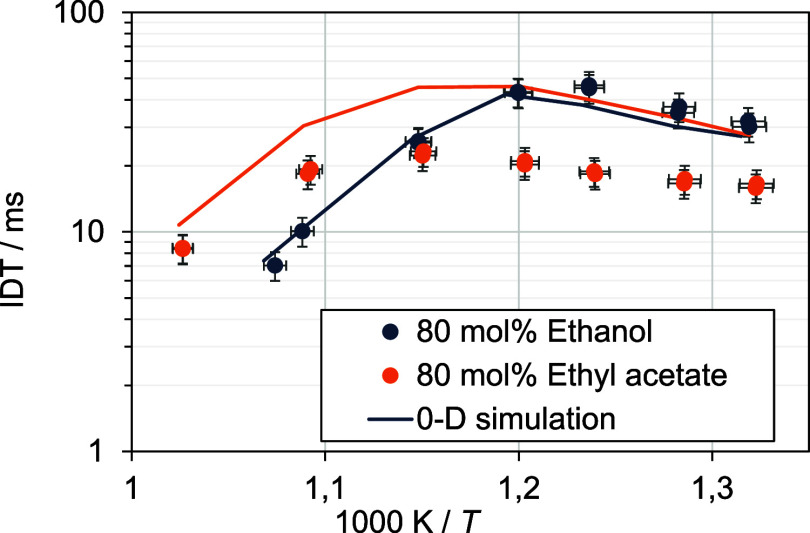
RCM ignition delay times at ϕ = 0.5, 17.5 bar, and air-like dilution for blends with 20 vol % *n*-heptane.

Using the RCM, the purely chemical ignition delay times were obtained. The IDT measurements for blends with 30 vol % *n*-heptane and 20 vol % *n*-heptane are shown as symbols, respectively, in Figures 9 and 10. Simulations obtained with corresponding volume ratio profiles and the merged blend mechanism are shown as solid lines in the figures. The end-of-compression temperature varies from 756 to 983 K at a constant end-of-compression pressure of 17.5 bar. The equivalence ratio (ϕ) was 0.5 and the dilution 3.76 with a mixture of 50% CO_2_ and 50% N_2_.

Even for a small amount of only 20/30 vol % *n*-heptane, the negative temperature coefficient (NTC) regime is well pronounced for both blends. The local maxima of the NTC regime shifts to higher temperatures with a higher *n*-heptane fraction: for ethanol from 809 to 834 K and for ethyl acetate from 869 to 874 K. The behavior of ethanol blends being more reactive than ethyl acetate blends is observed inside the NTC regime, e.g., for the 30 vol % *n*-heptane blend from 758 to 893 K. At higher temperatures, the trend flips again, and the ethyl acetate blend is more reactive than the ethanol blend. The same is indicated for the low-temperature end of the NTC regime. The IDT prediction of both ethanol blends is within the experimental uncertainties; meanwhile, the prediction of the ethyl acetate blends can only capture the trend but not the exact values. The overprediction of the ethyl acetate blend IDTs becomes less at the borders of the NTC regime, see 976 K (1000*K*/*T* < 1.02) at 30 vol % *n*-heptane and 758 K (1000 *K*/*T* > 1.32) at 20 vol % *n*-heptane. In summary, the ethyl acetate model still needs some refinement in future, but as it is capable to predict most of the trend, it is assumed that the model is accurate enough to capture the most important reaction pathways.

### Effect of Mixing Ratio on Physical Properties

**Figure 11:**
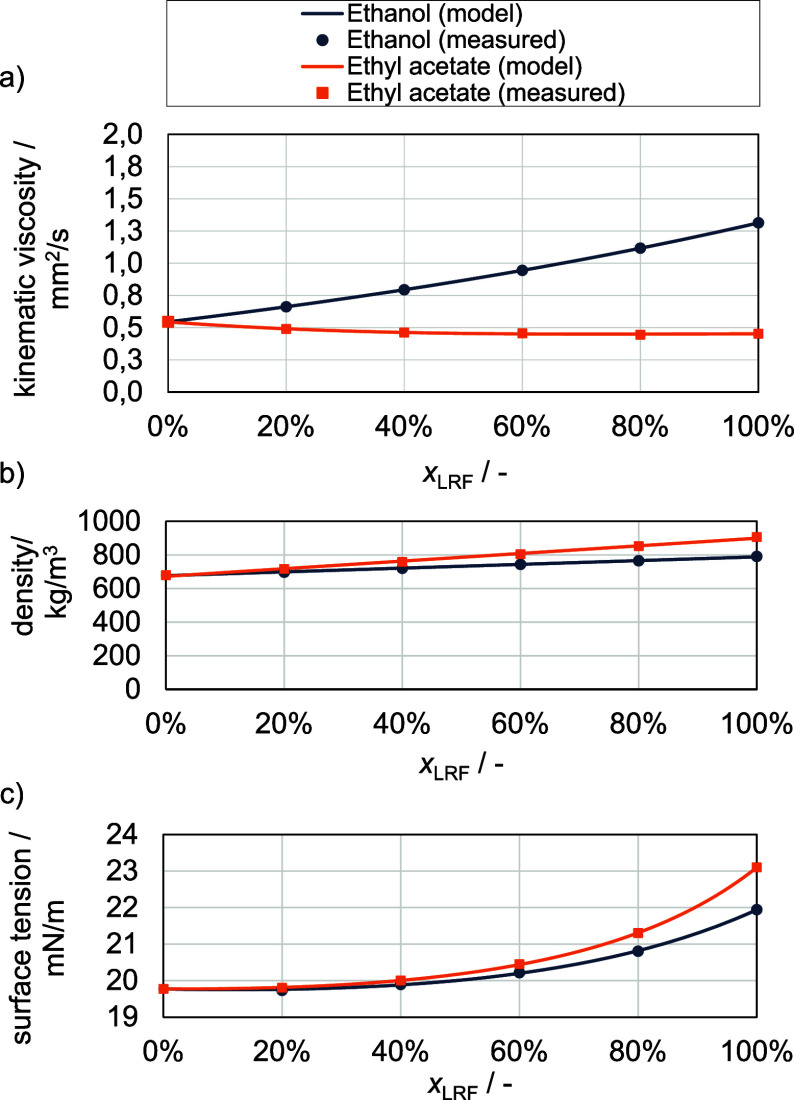
Measurement results for physical fluid properties.

In Figure 11, the results for the physical fluid properties of the binary fuel mixtures are shown. For each property, the measured values as well as interpolated data based on different mixing models are depicted. In case of the kinematic viscosity (Figure 11a) the model according to Redlich–Kister is applied.^12,14^ For the interpolation of density (Figure 11b), a linear approach is used, and for the interpolation surface tension (Figure 11c), a model according to Connors and Wright^13^ is used. Each model is a based on two variable parameters and was fitted against the measured data.

In Table 2, the coefficients of determination *R*^2^ and  are depicted. The high values indicate a good agreement between measured values and the used mixing rules. Therefore, a noncontinuous behavior of physical fluid properties depending on the mixing ratio can be excluded as reason for the observed deviation of ignition delay. Furthermore, the values for the two blends show no high deviations. Especially, the measured surface tension deviates only slightly for both mixtures. Table 1

**Table 1:** Evaluation of Dimensionless Numbers

Reynolds number	
Ohnesorge number	
Weber number	

**Table 2:** Performance of Regression Models

	Ethanol/*n*-Heptane		Ethyl acetate/*n*-Heptane	
	*R*^2^		*R*^2^	
Viscosity	1.0000	1.0000	0.9927	0.9908
Density	0.9975	0.9969	0.9960	0.9950
Surface tension	0.9996	0.9995	1.0000	0.9999

### Injection Characteristics

In this section the atomization behavior of various fuel blends is put into perspective using the Ohnesorge diagram. Additionally, the results gained by the use of the injection progress indicator will be presented.

#### Effect of Mixing Ratio on Dimensionless Numbers

**Figure 12:**
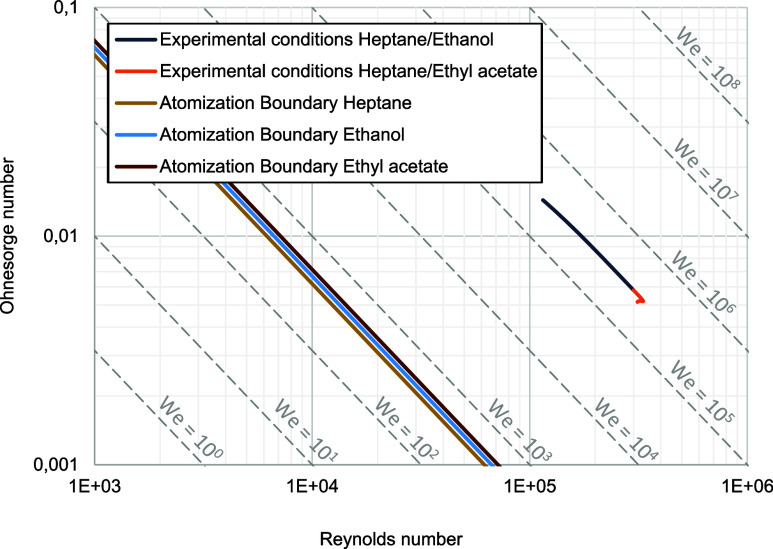
Ohnesorge diagram based on determined fluid properties.

The corresponding Ohnesorge diagram for the chamber pressure *P*_*ch*_ = 17.5 bar and a temperature of *T*_*ch*_ = 853 K, is depicted in Figure 12. The resulting air density of ρ_*air*_ = 7.1  is calculated with help of the ideal gas law. Due to deviating densities of the pure fuels, a different Weber number and boundary line for the atomization regime is obtained for each mixing ratio. In Figure 12, only the boundaries for pure ethanol, ethyl acetate, and *n*-heptane are depicted (lines in the left half). The boundaries for the binary mixtures would be located between these lines.

Both the Ohnesorge number and Reynolds number differ greatly depending on the mixing ratio for the two blend systems. However, the calculated Weber numbers range in a relatively small span between *We* = 3.03 × 10^6^ and 2.60× 10^6^ for the ethyl acetate/*n*-heptane blend and *We* = 3.03 × 10^6^ and 2.73 × 10^6^ for the ethanol/*n*-heptane blend, respectively. Therefore, a very similar spray behavior can be assumed for each fuel blend independent from used fuels and the mixing ratio. It can also be seen that the mixtures are clearly in the range of atomization, since the calculated Weber numbers are nearly 3 orders of magnitude larger than Weber numbers for the boundaries. Therefore, the influence of the spray behavior on the ignition delay times is probably negligible. The dimensionless numbers used only provide a qualitative view of atomization, which, however, fits very well to the experimental data and the results of the simulation.

#### Effect of Mixing Ratio on Injection Rate

**Figure 13:**
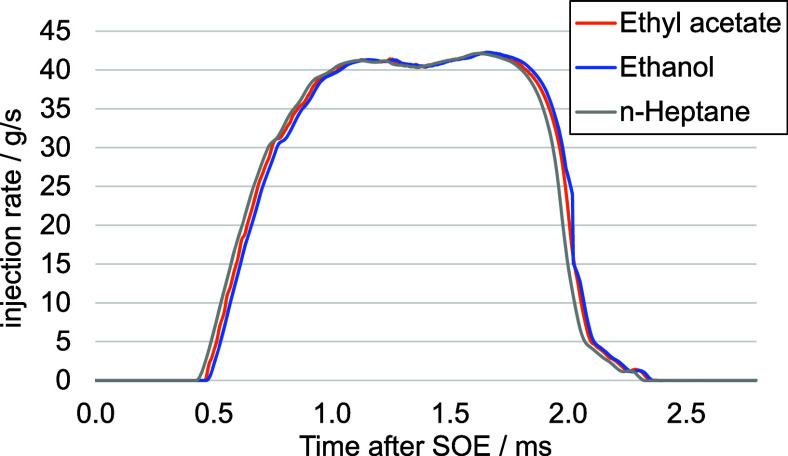
Injection rates for ethyl acetate, ethanol, and *n*-heptane.

The results from the investigations using the injection progress indicator revealed a lack of discernible disparities among the fuels examined. As depicted in Figure 13, the rate-of-discharge curves for *n*-heptane, ethanol, and ethyl acetate indicate that both the absolute quantity of fuel injected and the profiles of the injection curves show a striking similarity for all three fuels. This leads to the inference that substantial variations in ignition delay times are not to be ascribed to inherent influences of the fuel injection process. In essence, the methodology of fuel injection, along with associated attributes, does not appear to be the root cause of marked discrepancies in ignition delay times. The influence of factors such as density, viscosity, and surface tension on the results is seemingly negligible.

### Reaction Pathway Analysis

**Figure 14:**
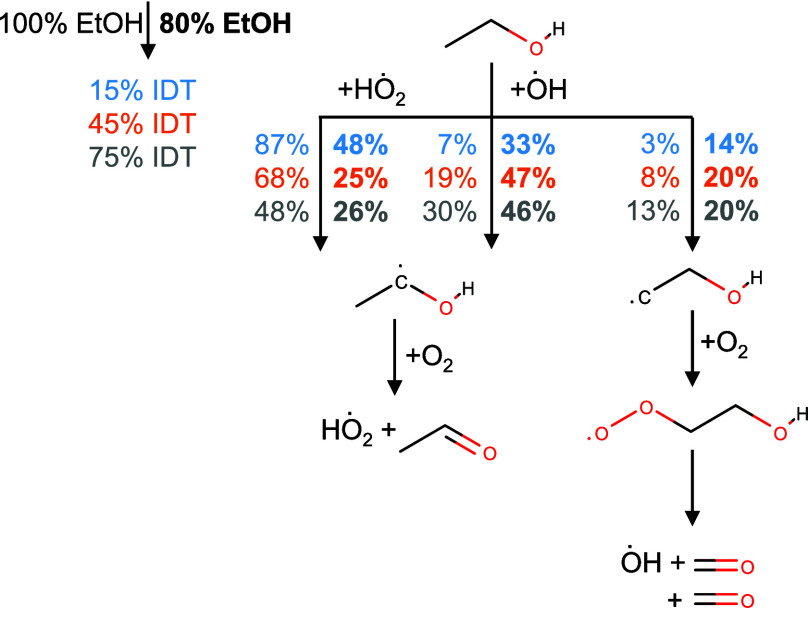
Rate of production analysis for neat ethanol and corresponding blends with 20 vol% *n*-heptane at *T*_ch_ = 800 K, *P*_ch_ = 17.5 bar, and ϕ = 0.5 in air.

**Figure 15:**
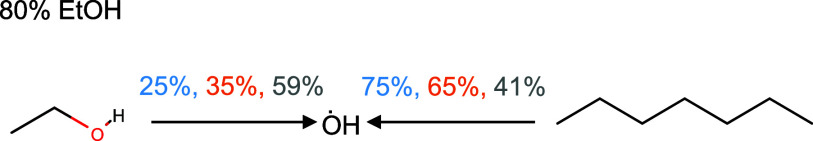
ȯH radical production analysis for blends with ethanol and 20 vol% *n*-heptane at 800 K, 17.5 bar, and ϕ = 0.5 in air.

**Figure 16:**
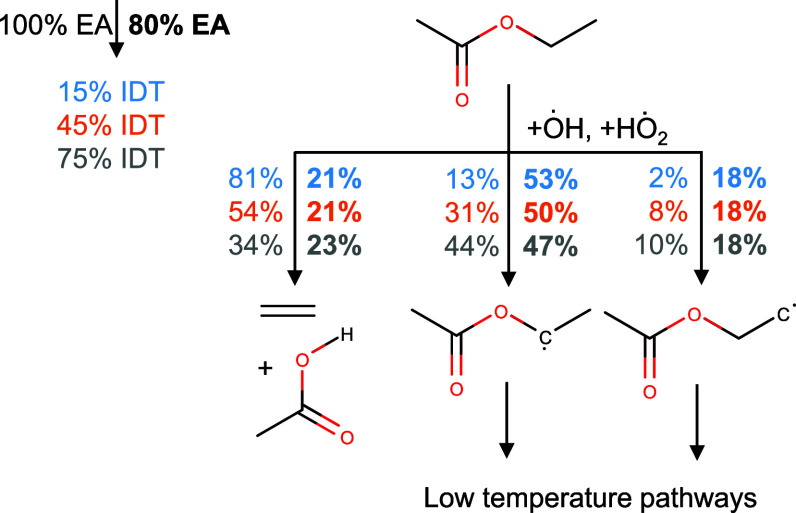
Rate of production analysis for pure ethyl acetate, and corresponding blends with 20 vol% *n*-heptane at 800 K, 17.5 bar, ϕ = 0.5 in air.

**Figure 17:**
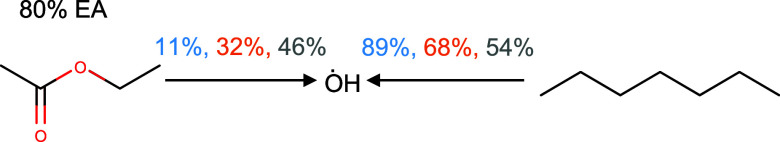
ȯH radical production analysis for blends with ethyl acetate and 20 mol % *n*-heptane at 800 K, 17.5 bar, and ϕ = 0.5 in air.

In the following, reaction pathway analyses have been performed for pure ethanol, pure ethyl acetate, an *n*-heptane blend with 80 vol% ethanol, and an *n*-heptane blend with 80 vol% ethyl acetate. The simulations have been performed as constant volume simulations at *T*_*ch*_ = 800 k, *P*_*ch*_ = 17.5 bar, and an equivalence ratio of ϕ = 0.5 in air. To observe reaction pathway changes over time, the pathway analyses have been performed at three timesteps that were normalized with the characteristic blend IDT (*IDT*_*EA*_ = 31.4 ms, *IDT*_*EtOH*_ = 33.8 ms) to 15% IDT, 45% IDT, and 75% IDT. The pathway analyses are shown in Figures 14 and 16. Furthermore, an H-atom flux analysis has been used to determine the ȯH production ratio of the two blending components (see Figures 15 and 17). To reduce the number of pathways leading to ȯH, only fluxes with 0.4% of the maximum appearing flux have been considered.

At the beginning of ethanol oxidation, at around 15% IDT, neat ethanol is primarily oxidized through H-atom abstraction (HAA) by Hȯ_2_, forming ethanol radicals with the radical position at the secondary carbon (see Figure 14). Later, the consumption shifts slowly toward HAA by ȯH, but the secondary radical is still favored (45% IDT and 75% IDT). Afterward, the reaction of O_2_ with the secondary radical leads to the formation of acetaldehyde and HO_2_ via a concerted elimination. Within the blend, *n*-heptane acts as a ȯH producer due to its strong low-temperature chemistry,^29^ which can be observed in the ȯH production flux analysis (see Figure 15). High-temperature reaction channels, which can lead to the production of ȯH radicals by the fission of H atoms and the subsequent reaction with O_2_ by H + O_2_ = ȯH + ȯ, did not appear to be significant in the rate of production analysis at the studied temperature of 800 K.

Due to the higher H availability, all fluxes due to HAA by ȯH increase. This also enhances the importance of the primary ethanol radical. At 15% IDT, the consumption toward the primary radical is increased from 3% for pure ethanol to 14% in the blend. This radical then forms two formaldehyde molecules and one H radical after the O_2_ addition and a consecutive isomerization and decomposition. At 45% IDT, the ethanol consumption by ȯH is already increased to 67%, in contrast to the 25% by HO_2_. Nevertheless, the main consumption pathway of ethanol inside the *n*-heptane blend is still via the secondary radical even though the importance of the primary radical channel has increased.

For ethyl acetate, the competition between the decomposition that forms ethylene plus acetic acid and the HAA reactions over time is of great importance as depicted in Figure 16. In the pure ethyl acetate case, at 15% IDT, 81% of the ethyl acetate is consumed via the decomposition reaction, while only 8% is degraded via HAA by ȯH and 7% by HO_2_. Meanwhile the radicals continue with typical low-temperature chemistry pathways that produce more reactivity increasing ȯH radicals, the ethylene and acetic acid are limited to less reactive high-temperature pathways. Then, as the ignition process advances in time (45% IDT and 75% IDT), the branching ratio shifts more toward the HAA reactions. In contrast to this reaction path, the ethylene/*n*-heptane blend starts directly with a much higher amount of ȯH radicals that have been formed by the *n*-heptane (see Figure 16). At 15% IDT, only 21% of ethylene gets consumed by the decomposition reaction and 71% by the HAA reactions which themselves produce more ȯH radicals (see Figure 17). This branching ratio stays nearly the same for the whole ignition process.

In conclusion, the presence of approximately 10% to 30% *n*-heptane in ethanol or ethyl acetate causes the following changes in the reaction pathways of ethanol or ethyl acetate: 1. For ethanol, the consumption pathway via acetaldehyde remains the dominant channel, meanwhile the importance of formaldehyde has increased slightly; 2. For ethyl acetate, the branching ratio of its decomposition and consumption by HAA reactions has changed strongly in favor of the HAA, especially at the beginning of the oxidation. Due to this change, the reactivity-inhibiting effect of ethyl acetate decreases rapidly with an increasing amount of *n*-heptane. For high contents of *n*-heptane, the LRFs do not have a strong impact on the radical pool and the ethyl acetate blend shows a similar behavior as the ethanol blend.

## Conclusions

The investigation of ignition delay times (IDTs) of various fuels across different blending ratios revealed strong nonlinearities correlating with blend composition. To find an explanation for the significant differences in the observed ignition delay times, the physical and chemical properties of the different binary fuel mixtures were investigated in this work. Initially, physical properties such as viscosity, density, and surface tension were analyzed, aligning closely with established mixing rules in the literature. Subsequently, the injection dynamics of these fuel mixtures were characterized using an injection progression indicator, which indicated no significant differences that might affect IDT. Additionally, the viscosity, density, and surface tension data contributed to the calculation of dimensionless numbers to describe nozzle flow, where, again, no discontinuities were identified in the Weber numbers across all liquids and mixing ratios, suggesting that fluid mechanical effects are unlikely to account for the observed IDT irregularities. This hypothesis was further confirmed by supplementary measurements in a Rapid Compression Machine (RCM). The experimental data gained from the rapid compression machine have affirmed the model’s capacity to deliver highly accurate predictions for the ignition delay time in ethanol/*n*-heptane mixtures. Nevertheless, when applied to the ethyl acetate/*n*-heptane mixtures, the model consistently forecasts a prolonged ignition delay time, surpassing the experimental results. From this, it was concluded, that the ethyl acetate model still needs refinements to fully understand the blending effect observed in this study. Contrarily, findings underscored the inadequacy of directly correlating the reactivity of a pure component to that of its blend. Notably, the investigation highlighted the role of a modified radical pool during ignition, significantly influencing primary reaction pathways and, consequently, affecting the radical pool itself. In the specific context of this study, it was noted that the addition of small amounts of *n*-heptane to a blend renders ethanol more advantageous over ethyl acetate as a knock-resistant fuel, despite the reverse being true for the pure components.

## Outlook

Part of the following research will be the refinement of the used models in order to enable more accurate predictions of the blending behavior. The nonlinear responses observed in the ignition delay times across different fuel blends, indicative of complex underlying interactions, underscore the necessity for a comprehensive investigation into these phenomena. Future studies should prioritize the advancement of the existing models to more accurately reflect the nuanced shifts in reactivity observed within these mixtures.

A profound comprehension of the chemical dynamics at play, particularly the shifts in reactivity prompted by blending, necessitates an exhaustive exploration of the combustion chemistry inherent to blends of *n*-heptane with low-reactive fuels (LRFs). This exploration should extend to a detailed examination of reaction pathways, the emergence of intermediates, and the genesis of radical species. Such an approach promises to shed light on the specific chemical processes that lead to the observed changes in reactivity.

Looking forward, the deployment of fuel blends tailored for optimized reactivity in engine environments represents a critical area of investigation. Assessing the real-world implications of these optimized blends on engine efficiency, power output, and emissions will provide a tangible measure of their potential impact. This practical application of research findings is essential for validating the theoretical models and simulations that advance the understanding of fuel blend reactivity and its implications for engine performance. Through these focused efforts, the study aims to contribute significantly to the development of fuel blends that are not only more efficient but also environmentally sustainable, aligning with the global imperative for cleaner combustion technologies.

## Supporting Information Available

Experimental results and mechanism (ZIP)
